# Mobilization of Genomic Islands of *Staphylococcus aureus* by Temperate Bacteriophage

**DOI:** 10.1371/journal.pone.0151409

**Published:** 2016-03-08

**Authors:** Bo Youn Moon, Joo Youn Park, D. Ashley Robinson, Jonathan C. Thomas, Yong Ho Park, Justin A. Thornton, Keun Seok Seo

**Affiliations:** 1 Department of Basic Sciences, College of Veterinary Medicine, Mississippi State University, Mississippi State, MS 39762, United States of America; 2 Department of Microbiology, College of Veterinary Medicine and BK21 Program for Veterinary Science, Seoul National University, Seoul, 151–742, South Korea; 3 Department of Microbiology and Immunology, University of Mississippi Medical Center, Jackson, MS 39216, United States of America; 4 Department of Biology, University of Bolton, Bolton, Greater Manchester, BL3 5AB United Kingdom; 5 Department of Biological Sciences, Mississippi State University, Mississippi State, MS 39762, United States of America; Institut National de la Recherche Agronomique, FRANCE

## Abstract

The virulence of *Staphylococcus aureus*, in both human and animal hosts, is largely influenced by the acquisition of mobile genetic elements (MGEs). Most *S*. *aureus* strains carry a variety of MGEs, including three genomic islands (νSaα, νSaβ, νSaγ) that are diverse in virulence gene content but conserved within strain lineages. Although the mobilization of pathogenicity islands, phages and plasmids has been well studied, the mobilization of genomic islands is poorly understood. We previously demonstrated the mobilization of νSaβ by the adjacent temperate bacteriophage ϕSaBov from strain RF122. In this study, we demonstrate that ϕSaBov mediates the mobilization of νSaα and νSaγ, which are located remotely from ϕSaBov, mostly to recipient strains belonging to ST151. Phage DNA sequence analysis revealed that chromosomal DNA excision events from RF122 were highly specific to MGEs, suggesting sequence-specific DNA excision and packaging events rather than generalized transduction by a temperate phage. Disruption of the *int* gene in ϕSaBov did not affect phage DNA excision, packaging, and integration events. However, disruption of the *terL* gene completely abolished phage DNA packing events, suggesting that the primary function of temperate phage in the transfer of genomic islands is to allow for phage DNA packaging by TerL and that transducing phage particles are the actual vehicle for transfer. These results extend our understanding of the important role of bacteriophage in the horizontal transfer and evolution of genomic islands in *S*. *aureus*.

## Introduction

Genetic variation of bacteria can be achieved through mutations, rearrangements and horizontal gene transfers and recombinations. Increasing genome sequence data have demonstrated that, besides the core genes encoding house-keeping functions such as essential metabolic activities, information processing, and bacterial structural and regulatory components, a vast number of accessory genes encoding antimicrobial resistance, toxins, and enzymes that contribute to adaptation and survival under certain environmental conditions are acquired by horizontal gene transfer of mobile genetic elements (MGEs) [1, 2]. Mobile genetic elements are a heterogeneous group of molecules that include plasmids, bacteriophages, genomic islands, chromosomal cassettes, pathogenicity islands, and integrative and conjugative elements [2–4]. Genomic islands are relatively large segments of DNA ranging from 10 to 200 kb often integrated into tRNA gene clusters flanked by 16–20 bp direct repeats [3]. They are recognized as discrete DNA segments acquired by horizontal gene transfer since they can differ from the rest of the chromosome in terms of GC content (%G+C) and codon usage [3, 5].

*Staphylococcus aureus* is a major pathogen that colonizes the skin and mucous membranes of humans and animals, causing diseases ranging from mild skin infections to severe invasive diseases such as necrotizing pneumonia, infective endocarditis, and osteomyelitis [6–8]. The pathogenicity of this bacterium is largely influenced by the virulence genes carried on MGEs [9]. Three types of genomic islands (νSaα, νSaβ, νSaγ) are known in *S*. *aureus* [2]. Each type of genomic island is polymorphic in gene content but conserved within strain lineages [10–12]. Genomic islands are not as competently mobile as other MGEs, due to the lack of typical genetic elements required for or indicative of mobilization such as integrases, excisionases, terminases, and associated repeat sequences [13, 14]. While efficient mobilization of SaPIs by temperate helper phages has been well documented [15, 16], direct evidence indicating the mechanism of genomic island mobilization has not been well established. Previously, we reported the transfer of νSaβ by temperate phage ϕSaBov, which integrated immediately adjacent to the νSaβ [17]. The induction of ϕSaBov by mitomycin C generated transducing particles harboring overlapping segments of νSaβ in circular and linear forms of phage DNA, which appeared to be followed by sequential integration and homologous recombination events, resulting in transfer of entire ϕSaBov and νSaβ [17]. Here we demonstrate, for the first time, phage-mediated transfer of genomic islands νSaα and νSaγ, which are remotely located from ϕSaBov. Our results also showed that the genetic background of the host and recipient strains impact the ability and efficiency of transfer of MGEs mediated by bacteriophages, suggesting the presence of MGE-specific mechanisms of excision and integration from the donor and the recipient strains, respectively, in concert with functions from bacteriophages.

## Materials and Methods

### Bacterial strains and growth conditions

All strains and plasmids used in this study are listed in Table 1. *Staphylococcus aureus* strains were cultured in tryptic soy broth (TSB) or agar (TSA) plates (Difco) supplemented with tetracycline (5 μg/mL, Sigma-Aldrich) as necessary. *Escherichia coli* were grown in Luria-Bertani (LB) broth and agar plates supplemented with ampicillin (100 μg/mL, Sigma-Aldrich) as necessary. Twenty nine bovine mastitis isolates and 22 human isolates were kindly provided by QIA (Quarantine inspection agency, South Korea) and Patrick Schlievert (University of Iowa), respectively.

**Table 1:** A list of strains and plasmids used in this study.

Strain	Description	Reference or source
*Staphylococcus aureus*		
RF122	Bovine isolate, CC151	26
RF122 *set*::*tetM*	Indicative strain for transfer of νSaα	This study
RF122 *hla*::*tetM*	Indicative strain for transfer of νSaγ	This study
RF122 *tst*::*tetM*	Indicative strain for transfer of SaPIbov1	This study
RF122 *mdr*::*tetM*	Indicative strain for transfer of SaPI122	This study
RF122 *int*::*tetM*	RF122 Δ*int*	[17]
RF122 *set*::*tetM*, *int*::*cat*	Indicative strain for transfer of νSaα in Δ*int* background, RF122 Δ*int set*::*tetM*	This study
RF122 *hla*::*tetM*, *int*::*cat*	Indicative strain for transfer of νSaγ in Δ*int* background, RF122 Δ*int hla*::*tetM*	This study
RF122 *terL*::*tetM*	RF122 Δ*terL*	[17]
RF122 Δ*terL* pMin164 *terL*	Complementation of Δ*terL*	This study
*Escherichia coli*		
DH5α	Cloning host of pMAD and pMin164	Life Technologies
Top10	Cloning host of pCR4	Life Technologies
Plasmid		
pMAD-CM	Generating deletion mutants	[17]
pMAD-tetM	*tetM* insertion for screening transduction	[17]
pCR4-TOPO	TA cloning vector	Life Technologies
pMin164	High copy number vector for complementation	[18]

### Phage induction and transduction

Cultures were incubated at 37°C with 200 rpm until reaching mid-exponential phase, and then mitomycin C (1 μg/mL, Sigma-Aldrich) was added. Cultures were incubated at 30°C with 80 rpm until clear lysis was observed. The lysates were sterilized with syringe filers (0.22 μm, Nalgene). A phage spot test and the plaque-forming unit (PFU) were determined by soft agar (0.5%) overlay method.

For transduction experiments, the recipient strains were cultured to mid-log phase and adjusted to approximately 2×10^7^ CFU/mL. A phage solution containing approximately 10^8^ PFU/mL was added to the culture, and incubated for 30 min at 30°C for the phage absorption, followed by addition of sodium citrate solution (100 mM, pH 4.5). After centrifuging at 4,000 rpm at 4°C for 15min, the pellet was suspended in sodium citrate solution and plated on TSA supplemented with tetracycline (5 μg/mL).

### Phage DNA extraction

Phage DNA purification was performed as previously described [17]. Briefly, heterogeneous chromosomal DNA of *E*. *coli* K-12 was added to the culture lysates induced by mitomycin C as a control for verification of DNase treatment previously described [17]. Excessive amounts of DNase I (Sigma-Aldrich, 100 unit each) were added to remove chromosomal DNA. The phage particles were precipitated with NaCl (0.5 M final concentration) and polyethylene glycol 8000 (10%, wt/vol), followed by ultracentrifugation at 100,000 × g for 1 h. Phage DNA was extracted from the pellets using DNeasy kit (Qiagen) according to the manufacturers’ instructions.

### PCR, outward PCR, and quantitative real time PCR

All primer pairs used in PCR, outward PCR, and quantitative real time PCR are listed in Table 2. PCR was performed to determine the presence of transducing phage particles harboring MGEs using primers specific to MGEs associated with strain RF122 including: νSaα (the *set* gene), νSaβ (the *lukE* gene), and νSaγ (the *hla* gene), SaPIbov1 (the *tst* gene), SaPI122 (the *mdr* gene), and ϕSaBov (the *int* gene). To estimate the frequency of transducing phage particles harboring MGEs, the absolute copy number of each MGE per nanogram of phage DNA was determined using quantitative real time PCR. Briefly, PCR products specific to MGEs (above) were cloned into pCR™4-TOPO® TA Vector (Life Technologies). Plasmids were quantified using a Nanodrop (Thermo Scientific) and the number of DNA copies/ng was calculated as described previously [19]. Quantitative real time PCR reaction was performed using SYBR green I master mix (Applied Biosystems) and a serial dilution of plasmid templates. Standard curves were generated by linear regression analysis calculating the slope, intercept, and correlation coefficient (R^2^) using Microcal OriginPro (Microcal origin, Version 7.5). Quantification of MGEs was calculated by interpolation the C_T_ from the standard curve.

**Table 2:** A list of primers used in this study.

Name	Sequences (5' to 3')
Detection of Specific virulence gene in MGEs	
intf	CATCACTGGTGGACGCTTTG
intr	AATGCATCGAGCGCTTTTTC
set1f	GACAGTAGGCAAGCTGCGAAT
set1r	TTTCTCTGCCGTCGATTGACT
lukEf	TTTTTTTCCATCAGGCGTAACA
lukEr	ACGAATGATTTGGCCATTCC
hlaf	GCACTTACTGACAATAGTGCC
hlar	TCGCCACCTATATATACCGTTTC
tstf	TGAATTTTTTTATCGTAAGCCCTTTG
tstr	GGAAATGGATATAAGTTCCTTCGCT
mdrf	CTTTTCCTAGAAGATACCGCAATGT
mdrr	CCCATCCTTCGTGCGTTAGT
qRT-PCR	
qintf	CATCACTGGTGGACGCTTTG
qintr	AATGCATCGAGCGCTTTTTC
qsetf	AGACAAGAACGCACGCCTAAA
qsetr	TTATGGTTGGAGATTGTGGTGTGT
qlukEf	AGGTGGCAATGGCTCATTTA
qlukEr	TTGCTGAACCTGACGGACC
qhlaf	GGCCAGGCTAAACCACTTTTG
qhlar	GCTAATGCCGCAGATTCTGA
qtstf	TGAATTTTTTTATCGTAAGCCCTTTG
qtstr	GGAAATGGATATAAGTTCCTTCGCT
qmdrf	CTTTTCCTAGAAGATACCGCAATGT
qmdrr	CCCATCCTTCGTGCGTTAGT
rnaIIIf	TGAGTTATTAAGCCATCCCAACTTAA
rnaIIIr	AAAATACATAGCACTGAGTCCAAGGA
hlgbf	AGGTAAAATAACACCAGTCAGCGTAA
hlgbr	TGGTGCATAATCAACGACGTTT
Selective marker	
tetMf	GCGCGTCGACGATCAAGAAACAAAGGCAACCCA
tetMr	GCGCGAATTCTAGGACACAATATCCACTTGTAG
Allelic replacement of *set*, RF122 *set*::*tetM*	
setupf	GCGCGGATCCACGCCGAAAACTAAAGTGACA
setupr	GCGCGTCGACTGCTAA ACTTGCTTTCGCAAT
setdnf	GCGCGAATTCTTGAGTCTCTAAGAACGCCGA
setdnr	GCGCAGATCTAAAGACATCAAGGCCATGTGT
dsetr	TGCGTATAAACACCTGCGTCT
Allelic replacement of *hla*, RF122 *hla*::*tetM*	
hlaupf	GCGCGAATCCTTACCTCATATAGTGTCATG
hlaupr	GCGCGGTCGACGAAAGGTACCATTGCTGGTC
hladnf	GCGCGGAATTCGTCAAATTAGAATATTGCAG
hladnr	GCGCGAGATCTAATGCCTATAACTAA AAACC
dhlar	AATGAATCCTGTCGCTAATGCC
Allelic replacement of *tst*, RF122 *tst*::*tetM*	
tstupf	GCGCGTCGACACCAATGCGGCAGTCGGTGAT
tstupr	GCGCACGCGTATTGGAAAATAACAATGAATGACGGA
tstdnf	GCGCGAATTCCACTACTATACCAGTCTAGCAAAT
tstdnr	GCGCCCCGGGGTGTACCAACATCTTTAATTTCTTCA
dtstr	AGTTCTATTGGAGTAGGTAATTTTTCAG
Allelic replacement of *mdr*, RF122 *mdr*::*tetM*	
mdrupf	GCGCGTCGACTAAACCTTAAACCCTCTAATTCAGT
mdrupr	GCGCACGCGTAGGAGTACTCATAACAGGTGTCGTTA
mdrdnf	GCGCGAATTCTCTTAGATACTCCTCTTTGGTT
mdrdnr	GCGCCCCGGGAATATTCGGAATAGGCTCGCAG
dmdrr	TGGCCATAATCGCGCCAACGA
Generating integrase knock-out strain, RF122 Δ*int*	
Intupf	GCGCGGATCCGCTCCTTTACGGAGCTTTAA
Intupr	GCGCGTCGACAATAAGGGTAGGCGAGCTAC
Intdnf	GCGCGAATTCGCATATCTTGGGAACGTTTC
Intdnr	GCGCAGATCTAACAGAGAACATGTTGCTAC
Allelic replacement of *hlgB*, RF122 *hlgB*::*tetM*	
hlgBupf	GCGCGGATCCCATTCGTGCAATCGGTTACC
hlgBupr	GCGCGTCGACAGCTAATCGATTTAGAATAG
hlgBdnf	GCGCGAATTCGGCTTTGTGAAACCTAATCC
hlgBdnr	GCGCAGATCTGGTCGTCACAATTACTGTG
dhlgBr	AATGGCAGTATTACTAAG
Generating terminaseL knock-out strain, RF122 Δ*terL*	
Terupf	GCGCGGATCCTGTCAACATGGCTTTTTCTG
Terupr	GCGCGTCGACTTGCTGAGGGTCTTGTGTTC
Terdnf	GCGCGAATTCCTTTCCGACCACGGGTTAA
Terdnr	GCGCAGATCTACGAAAGTTTGCCGGAAATA
Outwarding PCR	
pself	AGCGGTGTGATTCTGGTGAAT
p0342r	TGGCGCACTCATCAAAGAGT
pSAB1912f	TGG AAG AGA TTT TAT AAC TAA TTT TG
pSaPI122r	CAG TGG GGA CAC CTG TGT AA
pIntf	CGAGATTTAACGAGGGATAGG
p1702r	TTGACACTAGCTTTCCGTTG
Verification of chromosomal DNA contamination in phage DNA preparation	
waaQf	TAAAGGTGCGGGAACTTTCG
waaQr	AAGCGAGATCATCTGCCGAG
Complementation of *terL*	
tercompf	GCTAGGATCCATCGGACTCCGTCCCGTCAT
tercompr	GCTAGAATTCAGACTACAAAGAGAATCCCG

### Allelic exchange constructs

All PCR primer pairs used in allelic exchange constructs were listed in Table 2. The gene deletion mutants and the insertion of tetracycline resistance gene marker for screening transduction events were generated by allelic exchange using a modified pMAD-CM and pMAD-*tetM* temperature-sensitive shuttle vector system [17], respectively. Briefly, the gene fragment of upstream and downstream of target gene were cloned in pMAD-CM and pMAD-tetM. Resulting plasmids were electroporated to *E*. *coli* DH5α, and then to strain RF122. RF122 harboring the constructed plasmid were cultured at 43°C (non-permissive temperature for the replication of pMAD) to promote the first homologous recombination, followed by culturing 37°C to promote the second recombination, resulting in allelic exchange.

### Phage and bacterial genomic DNA sequencing and analysis

Phage capsid DNA was isolated as described above, and bacterial genomic DNA was isolated with a DNeasy kit according to the manufacturer’s instructions (Qiagen). The dsDNA was quantified with a Qubit HS Assay Kit (Invitrogen). Indexed, paired-end libraries were prepared with a Nextera XT DNA Sample Preparation Kit (Illumina). Libraries were cleaned with 1.2× AMPure XP beads (Agencourt) and sequenced using a 300 cycle MiSeq Reagent Kit v2 on an Illumina MiSeq instrument (Illumina). CLC Genomics Workbench v6 software was used to trim and filter reads for quality and to assemble reads *de novo*. Recombined regions among the RF122 donor (GenBank NC_007622), CTH96 recipient, and transductants were identified through local alignments.

## Results

### Transducing phage particles induced from RF122 harbor mobile genetic elements (MGEs)

**Fig 1:**
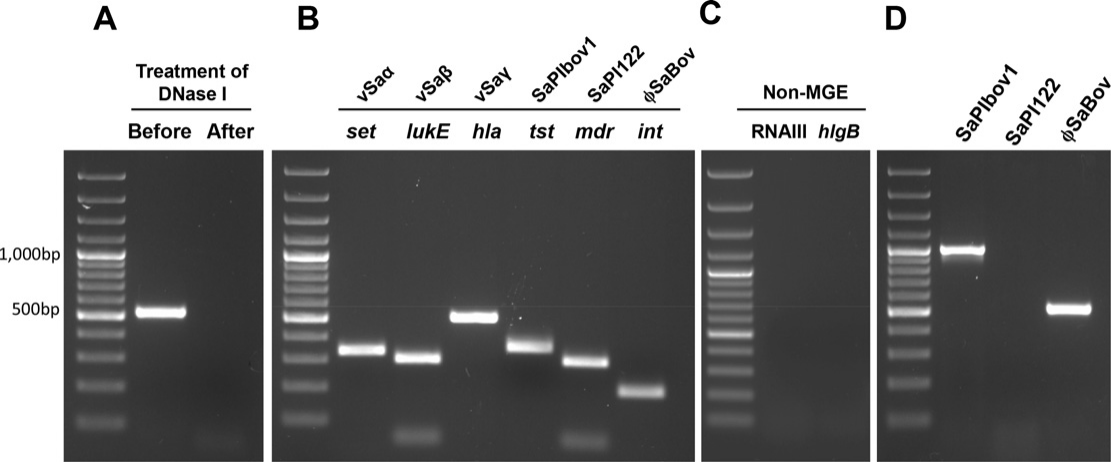
The presence of MGEs in transducing particles induced from the RF122 strain. (A) Verification of chromosomal DNA removal in preparation of phage DNA by adding exogenous chromosomal DNA of *E*. *coli*, followed by the treatment with DNase. The presence of MGEs in the phage DNA from transducing particles induced from the RF122 strain was analyzed by PCR amplification using primers specific to (B) MGEs; νSa (*set*), νSaβ (*lukE*), νSaγ (*hla*), SaPIbov1 (*tst*), SaPI122 (*mdr*), ϕSaBov (*int*) and (C) non-MGE (*rnaIII*, *hlgB*). (D) Outward PCR analysis of circularization of MGEs flanked with the direct repeat sequence; SaPIbov1, SaPI122, and ϕSaBov.

To investigate the possibility that transducing phage particles induced from strain RF122 harbor genes uniquely associated with MGEs, PCR was performed using phage DNA extracted from RF122 following mitomycin C treatment. To ensure the removal of RF122 chromosomal DNA, exogenous *E*. *coli* chromosomal DNA was added to the phage-induced lysates and treated with an excessive amount of DNase I. A PCR targeting the *waaQ* gene of *E*. *coli*was performed to confirm the removal of chromosomal DNA in the phage DNA preparation (Fig 1A). Results of PCR revealed that transducing phage particles induced from RF122 harbor genes associated with MGEs including genomic islands νSaα (the *set* gene), νSaβ (the *lukE* gene), and νSaγ (the *hla* gene), SaPIbov1 (the *tst* gene), SaPI122 (the *mdr* gene), and ϕSaBov (the *int* gene) (Fig 1B). The genes not belonging to MGE (*hlgB* and *rnaIII*) were not detected (Fig 1C). The SaPIbov1and ϕSaBov bordered with direct repeat sequences generated amplification products in outward PCR indicating a circular form of phage DNA. Sequencing of amplification products further confirmed these findings (data not shown). The SaPI122, not bordered with direct repeat sequence did not generate an amplification product suggesting a linear form of phage DNA, as expected for *cos*-type of sticky-end linear molecules (Fig 1D).

**Fig 2:**
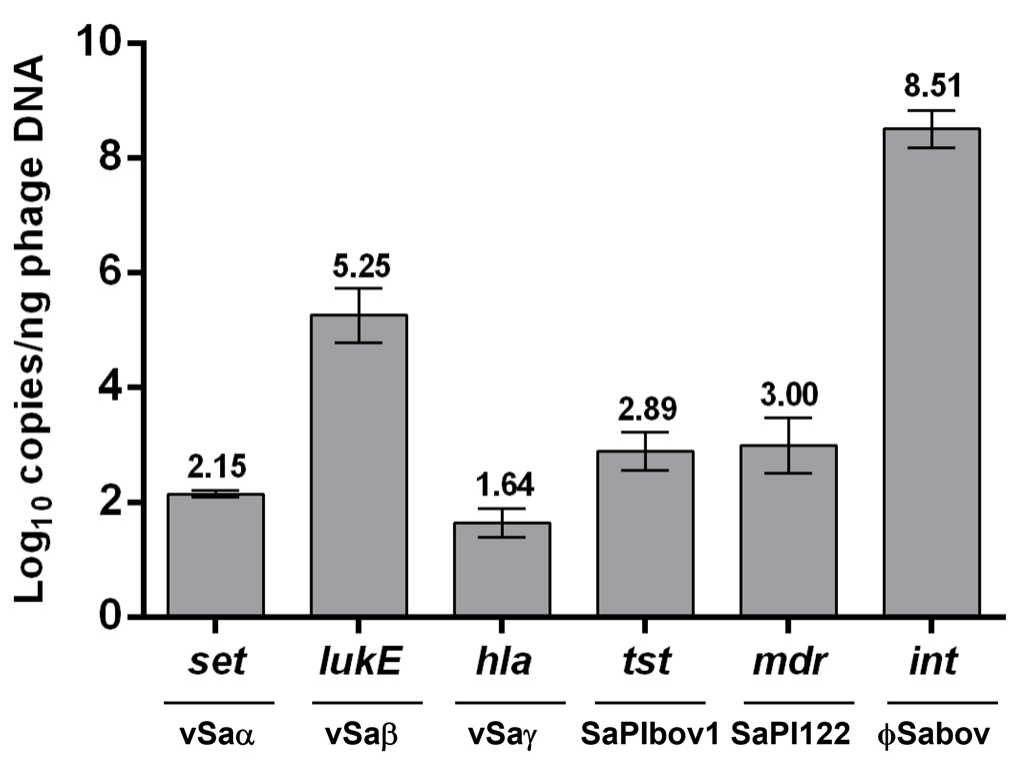
Estimation of the absolute copy number of MGEs in phage DNA using quantitative real time PCR.

To estimate the frequency of transducing phage particles harboring MGEs, the absolute copy number of each MGE per nanogram of the phage DNA was interpolated from a standard curve generated using quantitative real time PCR (data not shown). The copy number of the ϕSaBov was the highest (8.51 Log_10_ copies/ng phage DNA), followed by νSaβ (5.25 Log_10_ copies/ng phage DNA). The copy numbers of νSaα, νSaγ, SaPIbov1, and SaPI122 were lower than the ϕSaBov and νSaβ, and ranged between 1.64–3.00 Log_10_ copies/ng phage DNA (Fig 2).

### Sequence analysis of phage DNA

**Fig 3:**
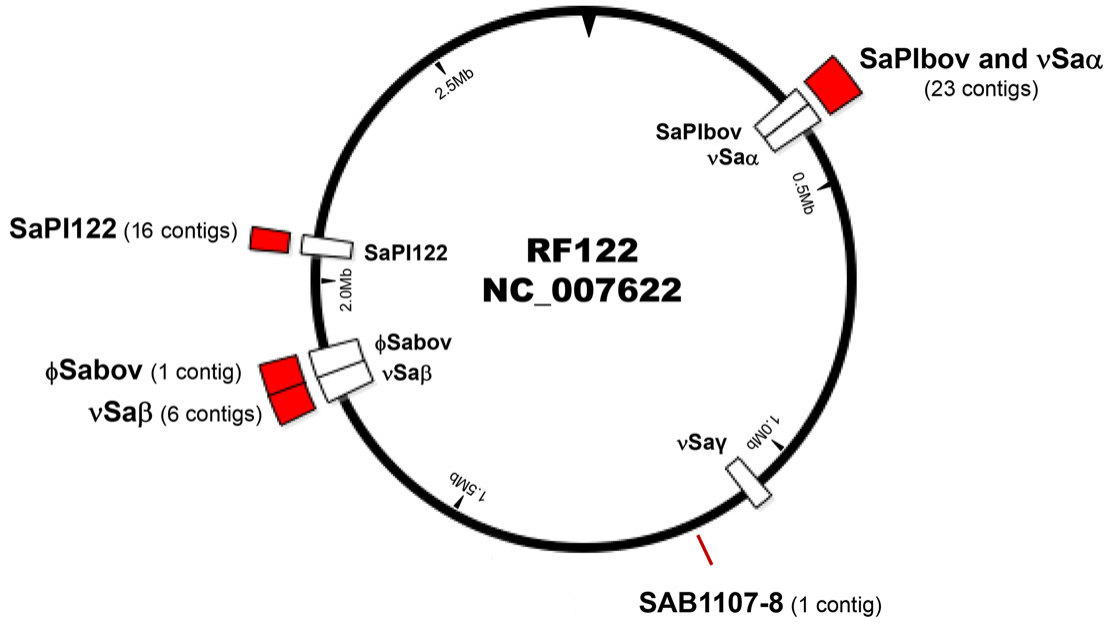
A schematic map of contigs determined from Illumina MiSeq analysis of phage DNA. A red box indicates the location and number of contigs detected from sequencing analysis of phage DNA. A white box indicates the location of MGEs (Sa,Sa, Saγ, SaPIbov1, SaPI122, ϕSaBov) present in the RF122 chromosome.

The DNA sequences from phage capsids were determined in order to identify and map the location of the RF122 chromosomal DNA packed into transducing phage particles. A total of 47 contigs (>500 bp) were assembled which clustered in MGEs including νSaα, νSaβ, SaPIbov1, SaPI122, and ϕSaBov, except for a single short contig at SAB1107-8 not known to be located on MGEs (Fig 3). The ϕSaBov was assembled in a single contig, while other MGEs contained multiple contigs ranging 6–23 contigs. A contig corresponding to νSaγ was not found in phage DNA sequencing, which might be due to the low copy number of the νSaγ regions as shown in quantitative real time PCR. These results suggest that the mechanism of excision and packaging of phage DNA is specific to MGEs, rather than a generalized transduction mechanism, which would result in random excision and packaging of host chromosomal DNA.

### Transfer of MGEs by transducing phage particles induced from RF122

**Fig 4:**
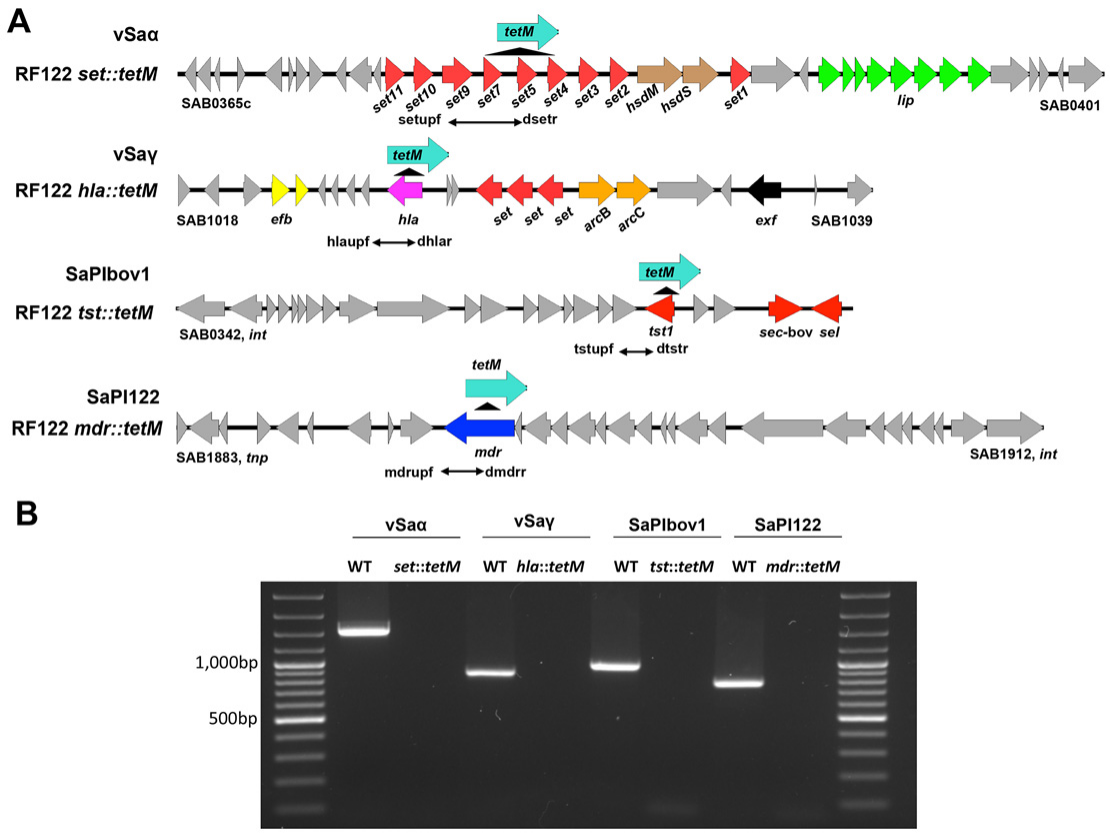
Schematic maps of the *tetM* gene insertion in the MGEs present in the RF122 strain. (A) The *tetM* gene was inserted into the MGEs present in the RF122 strain, including νSaα, νSaγ, SaPIbov1 and SaPI122, resulting RF122 *set*::*tetM*, RF122 *hla*::*tetM*, RF122 *tst*::*tetM*, and RF122 *mdr*::*tetM*, respectively. (B) The insertion of *tetM* was confirmed by PCR using primer sets designed to present a negative result in the insertional mutants, compared to the RF122 wild type strain (WT).

Results of PCR and phage DNA sequencing demonstrated the presence of phage particles harboring MGEs in the RF122 strain. To test the transfer of MGEs by these transducing phage particles to other *S*. *aureus*, the *tetM* gene, conferring tetracycline resistance, was introduced into the MGEs as depicted in Fig 4A by homologous recombination using a modified pMAD system. The insertion of the *tetM* gene was confirmed by PCR (Fig 4B). The transfer of MGEs was successful mostly in the ST151 lineage (Table 3). Other than ST151 strains, νSaα and SaPIbov1 were transferred only to ST1-SCCmecIV and ST398, respectively. Interestingly, among the ST151 strains, SaPIbov1 was only transferred to recipients (RF114, 38963, CI2135) which do not have SaPIbov1 in the genome. SaPIbov1 was not transferred to the recipients (CTH96, RF113, DS102) which have pre-existing SaPIbov1 in the genome. We also inserted the *tetM* gene into the non-MGE gene (*hlgB*) to test random excision and packaging of phage DNA as in true generalized transduction, but transduction was not observed even with the recipients belonging to the ST151 lineage (data not shown).

**Table 3:** Transduction frequencies of mobile genetic elements.

Recipient Origin	Recipient Genotypes	Strain			MGE	
			νSaα	νSaγ	SaPIbov1	SaPI122
^a^Bovine (29)	ST151 ^b^(6/6)	CTH96	^c^1.30×10^−6^	8.00×10^−7^	None	None
		RF113	1.65×10^−5^	7.00×10^−7^	None	2.43×10^−6^
		RF114	1.10×10^−6^	1.40×10^−6^	3.60×10^−7^	None
		38963	2.70×10^−6^	1.00×10^−6^	3.10×10^−7^	None
		CI2135	2.70×10^−6^	1.50×10^−6^	4.00×10^−8^	None
		DS102	3.50×10^−6^	None	None	None
	ST1 (0/3)		None	None	None	None
	ST188 (0/8)		None	None	None	None
	ST20 (0/5)		None	None	None	None
	ST72 (0/4)		None	None	None	None
	ST398 (1/3)	K31	None	None	4.5×10^−7^	None
Human (22)	ST1-SCC*mec*IV (3/7)	MW2	2.00×10^−8^	None	None	None
		MN KN	3.10×10^−7^	None	None	None
		C99-529	1.00×10^−8^	None	None	None
		C99-193	None	None	None	None
		MN Gary	None	None	None	None
		MN Ask	None	None	None	None
		MN MA	None	None	None	None
	ST8-SCC*mec*IV (0/7)		None	None	None	None
	ST36-SCC*mec*II (0/8)		None	None	None	None

^a^The number of tested strains is in parenthesis.

^b^The number of strains transduced with any MGE/the number of strains tested.

^c^Transduction frequencies (CFU/pfu)

**Fig 5:**
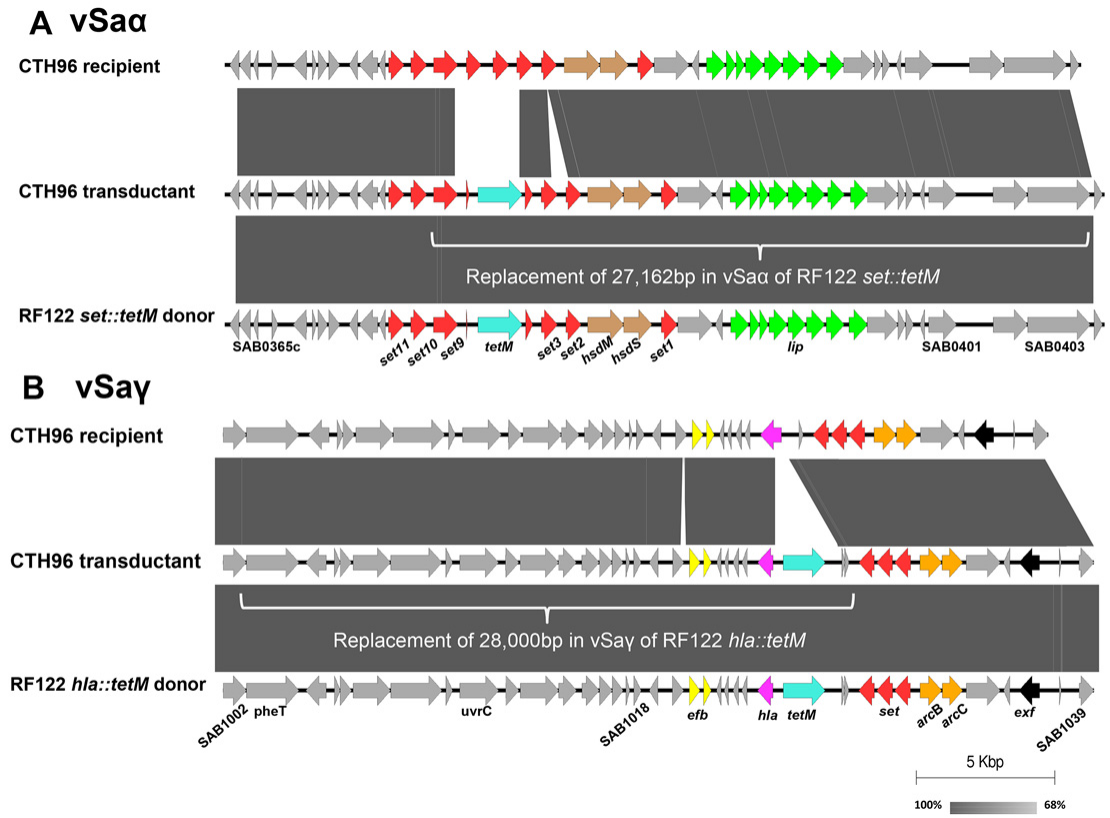
A schematic map of sequence alignments among RF122 (donor), CTH96 (recipient), and CTH96 transductants of phage induced from RF122 *set*::*tetM* (A) and RF122 *hla*::*tetM* (B). White brackets indicated the identical sequence between CTH96 transductant and RF122 donor, suggesting gene transfer from RF122 to CTH96. The shading between the entries represents the percent identity (BLASTn) from 68% (light gray) to 100% (dark gray) using Easyfig.

To further confirm the transfer of MGEs, a draft genome sequence of a recipient (CTH96) and transductants (CTH96 νSaα transductant and CTH96 νSaγ transductant) was determined. Based on single nucleotide polymorphisms (SNPs) and the *tetM* gene found in the CTH96 νSaα transductant, approximately 27,162 bp of νSaα was transferred from donor to recipient, ranging from SAB0378 to SAB0403 and including a part of the *set* gene locus, *hsdM*, *hsdS*, and the *lip* gene locus (Fig 5A). The sequence comparison of the CTH96 νSaγ transductant suggested that approximately 28,000 bp of νSaγ was transferred from donor to recipient, ranging from SAB1002 to SAB1029 and including phenylalanyl-tRNA synthetase (*pheT*), excinuclease ABC subunit C (*uvrC*), recombination and DNA strand exchange inhibitor protein (*mutS2*), ribonuclease III (*rnc*), succinate dehydrogenase (*sdh*), extracellular fibrinogen binding protein (*efb*), and the *hla* gene (Fig 5B).

### The role of integrase and terminase on ϕSaBov in the transfer of MGEs

**Fig 6:**
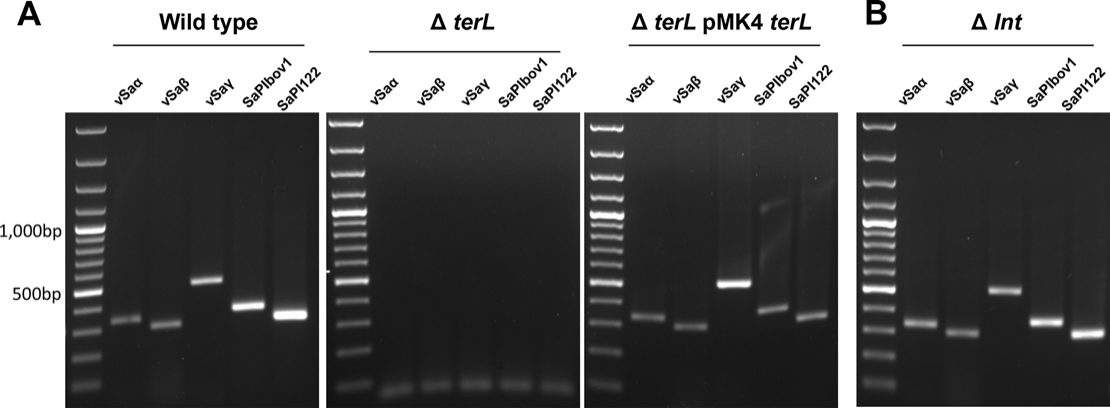
The roles of terminase large subunit (TerL) and intergrase (Int) on phage DNA excision and packaging. The terminase large subunit knock-out and integrase knock-out strains were generated from the RF122 strain by allelic replacement. The presence of MGEs in the phage DNA was analyzed by PCR with primers specific to each MGEs; νSaα (*set*), νSaβ (*lukD*), νSaγ (*hla*), SaPIbov1 (*tst*), and SaPI122 (*mdr*). A disruption of the TerL completely abolished the phage DNA packaging events. A complementation of TerL by pMin164 *terL* restored the phage DNA packaging events. (B) A disruption of the Int did not affect the phage DNA packaging events.

The integrase and terminase encoded in MGEs, including bacteriophages and SaPIs, are important for phage DNA excision, packaging, and integration, in concert with other factors derived from the donor or recipient [15]. However, the integrase and terminase were not present in the genomic islands νSaα and νSaγ. To test the involvement of the integrase (*int*) and terminase large subunit (*terL*) encoded by ϕSaBov in the transfer of these genomic islands, the *int* or *terL* gene was deleted in the RF122 strain. PCR analysis of phage DNA extracted from these strains showed that disruption of the *terL* gene completely abrogated phage DNA packaging, which could be restored by complementation of *terL* (Fig 6A). In contrast, disruption of the *int* gene did not affect DNA packaging (Fig 6B) nor the transfer of νSaα and νSaγ (Table 4). These results indicate that packaging of genomic islands in ϕSaBov is dependent on the terminase, but does not require integrase.

**Table 4:** The role of the integrase encoded in the ϕSaBov on transduction frequencies of MGEs.

	RF122		RF122Δ*int*	
Recipient strains	νSaα	νSaγ	νSaα	νSaγ
CTH96	^a^1.45×10^−6^	7.60×10^−7^	1.50×10^−6^	2.00×10^−7^
RF113	1.85×10^−5^	7.20×10^−7^	1.70×10^−6^	1.30×10^−7^
RF114	1.30×10^−6^	1.50×10^−6^	1.00×10^−6^	2.10×10^−7^
38963	2.50×10^−6^	1.20×10^−6^	2.00×10^−6^	1.50×10^−7^
CI2135	2.90×10^−6^	1.40×10^−6^	2.50×10^−6^	2.00×10^−7^
DS102	3.90×10^−6^	None	4.00×10^−6^	None

^a^Transduction frequencies (CFU/pfu)

**Fig 7:**
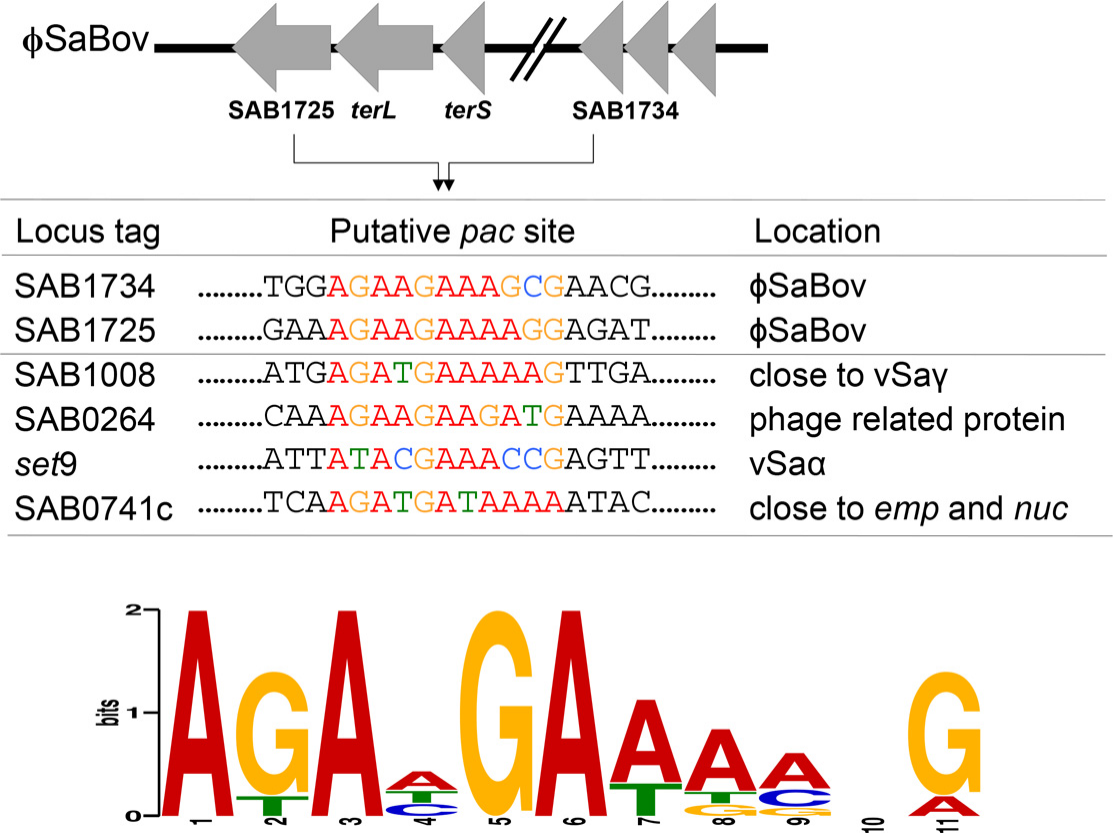
An 11 bp consensus sequence identified near to the *terL* gene using MEME (http://meme-suite.org/index.html) was conserved in MGEs packaged in the transducing phage particles. Putative *pac* site in MGEs. Sequence logos separated with colors indicated the frequencies scaled relative to the conservation at each position.

In *pac*-type phages, the terminase small subunit (TerS) recognizes and binds to the phage-specific packaging (*pac*) site typically located in or near to the *terL* gene that initiates a hetero-oligomer complex with TerL resulting in headful phage DNA packaging [15, 20, 21]. Analysis of phage DNA sequence showed an 11 bp consensus sequence present in ϕSaBov, νSaα, and νSaγ (Fig 7), suggesting that MGE-specific phage DNA packaging by ϕSaBov might result from the recognition of *pac* sites in the MGE by TerS that induce the headful packaging mechanism in concert with TerL and other factors in the ϕSaBov.

## Discussion

A transfer of MGEs by temperate phages requires a series of successful sequential events including excision from the host genome, packaging into transducing phage particles, transfer to a recipient and integration into the recipient genome. To investigate phage DNA excision events, phage DNA analysis using PCR and sequencing was performed. These results revealed that phage DNA excision events by ϕSaBov are highly specific to chromosomal locations of MGEs, suggesting a mechanism of sequence-specific excision events. It has been shown elsewhere that a typical *pac*-type phage DNA excision event is highly specific to the direct repeat sequence recognized by the integrase, excisionase, and terminase small unit [15, 21]. For MGEs, such as ϕSaBov and SaPIbov1, that are flanked by direct repeat sequences, phage DNA excision events occurred at the direct repeat sequence and formed a circular form of phage DNA. This process was likely controlled by their own integrase, excisionase, and terminases. For MGEs not flanked by direct repeat sequences, including νSaα and νSaγ, phage DNA excision events occurred at various locations within these genomic islands. νSaα and νSaγ do not possess annotated genes for excision events such as integrase, excisionase, and terminase, so phage DNA excision events in these MGEs might be controlled by other elements associated with SaPIs, transposases, integrative and conjugative elements in the host background. To support this possibility, the RN4220 harboring ϕSaBov, which has a different background of integrase, transposases, and integrative and conjugative elements than RF122 and lacks MGEs such as SaPIbov1, SaPI122, did not generate transducing phage particles harboring MGEs (data not shown).

As determined by PCR and DNA sequence analysis of phage DNA, transduction events are specific to MGEs rather than the random events that are expected from generalized transduction. A recent study demonstrated that the TerS encoded by SaPIs induced sequence-specific excision and packaging of phage DNA at unlinked chromosomal segments [15]. In our current study, sequence analysis of MGEs present in transducing particles revealed potential consensus sequences in these MGEs which might mediate DNA excision/packaging events by TerS encoded in the ϕSaBov, SaPIbov1 or SaPI122. Currently, the role of TerS and these potential consensus sequences on horizontal gene transfer is under investigation.

A higher frequency of MGE transfer by ϕSaBov was observed with recipient strains belonging to ST151 than with other strain lineages. These results suggest that the host specificity of phage might be a primary barrier in the transfer of MGE by phage. The host specificity of phage is expected to be determined by interactions between the tail proteins of phage and the target molecules in recipient cells such as proteins and wall teichoic acid (WTA) which were generally conserved in the core genome of the same or related lineages of strain [22–25]. As such, strains belonging to ST151 lineage commonly express receptors to ϕSaBov and thereby become more susceptible to transduction. Interestingly, SaPIbov1 was not transferred to ST151 recipients that already possess SaPIbov1 in the genome. This observation is not likely superinfection immunity due to lysogenic phages since phage was not induced by mitomycin C treatment in these recipient strains [26]. It is possible that the conserved transcriptional regulators in the SaPIs, such as *stl* and *str* [27, 28], might negatively regulate transcription of elements required for the integration of the SaPIbov1.

To confirm the transfer of MGEs by ϕSaBov, a draft genome sequence of the transductants for νSaα and νSaγ was determined. The extent of the transfer of MGEs was estimated by comparing single nucleotide polymorphisms and the presence of the *tetM* gene among donors, recipients, and transductants. Based on these comparisons, it was hypothesized that linear phage DNA segments harboring parts of νSaα and νSaγ were transferred, rather than a transfer of a single contig harboring entire νSaα or νSaγ. This result supports findings that the overall structure of genomic islands can vary as a result of “plug and play” type of recombinational replacements [29]. To our knowledge, this is the first report to demonstrate a mobilization of νSaα and νSaγ. Although the *hla* gene was largely thought to have evolved with the core chromosome of *S*. *aureus*, recombination events are evident in some strains [30, 31]. Our results suggest one mechanism by which the *hla* gene could be horizontally transferred among *S*. *aureus* strains.

Our previous study demonstrated that the *int* gene encoded by the ϕSaBov is required for integration of phage DNA harboring ϕSaBov and νSaβ into recipient genome, but not for phage DNA excision events of ϕSaBov and νSaβ [17]. Further, the *terL* encoded in the ϕSaBov is required for packaging phage DNA of ϕSaBov and νSaβ [17]. Similarly, in this study, the *int* gene encoded by ϕSaBov is not required for phage DNA excision events in νSaα and νSaγ nor for the integration into recipient genome. However, the *terL* is essential for packaging into transducing phage particles. These results suggest the major role of temperate phage in transfer of νSaα and νSaγ is to package DNA and shuttle it via transducing phage particles.

Although it has long been acknowledged that bacteriophages contribute the horizontal transfer of MGEs by generalized and specialized transduction, the causal link to the transfer of genomic islands by bacteriophages or other means has not been established. In this study, for the first time, we present experimental evidence demonstrating transfer of genomic islands by a temperate phage utilizing MGE-specific DNA excision events from the host genome and presumably recipient-derived factors for integration. These results represent the complexity of underlying mechanism of phage-bacterial interaction in horizontal gene transfer and extend our understanding of the important role of bacteriophage in the horizontal transfer and evolution of genomic islands in *S*. *aureus*.
